# Real-Time Monitoring of ATG8 Lipidation in vitro Using Fluorescence Spectroscopy

**DOI:** 10.21769/BioProtoc.4917

**Published:** 2024-01-05

**Authors:** Wenxin Zhang, Taki Nishimura, Sharon A. Tooze

**Affiliations:** 1 Molecular Cell Biology of Autophagy Laboratory, The Francis Crick Institute, London, UK; 2 Department of Biochemistry and Molecular Biology, Graduate School and Faculty of Medicine, The University of Tokyo, Tokyo, Japan; 3 PRESTO, Japan Science and Technology Agency, Tokyo, Japan

**Keywords:** Autophagy, In vitro ATG8 lipidation, Ubiquitin-like conjugation, Real-time ATG8 lipidation assay, Liposomes, Site-directed fluorescence, NBD, Fluorescence spectroscopy

## Abstract

Autophagy is an essential catabolic pathway used to sequester and engulf cytosolic substrates via a unique double-membrane structure, called an autophagosome. The ubiquitin-like ATG8 proteins play an important role in mediating autophagosome membrane expansion. They are covalently conjugated to phosphatidylethanolamine (PE) on the autophagosomes via a ubiquitin-like conjugation system called ATG8 lipidation. In vitro reconstitution of ATG8 lipidation with synthetic liposomes has been previously established and used widely to characterise the function of the E1 ATG7, the E2 ATG3, and the E3 complex ATG12–ATG5-ATG16L1. However, there is still a lack of a tool to provide kinetic measurements of this enzymatic reaction. In this protocol, we describe a real-time lipidation assay using NBD-labelled ATG8. This real-time assay can distinguish the formation of ATG8 intermediates (ATG7~ATG8 and/or ATG3~ATG8) and, finally, ATG8-PE conjugation. It allows kinetic characterisation of the activity of ATG7, ATG3, and the E3 complex during ATG8 lipidation. Furthermore, this protocol can be adapted to characterise the upstream regulators that may affect protein activity in ATG8 lipidation reaction with a kinetic readout.

Key features

• Preparation of ATG7 E1 from insect cells (Sf9 cells).

• Preparation of ATG3 E2 from bacteria (*E. coli*).

• Preparation of LC3B S3C from bacteria (*E. coli*).

• Preparation of liposomes to monitor the kinetics of ATG8 lipidation in a real-time manner.

**Graphical overview**


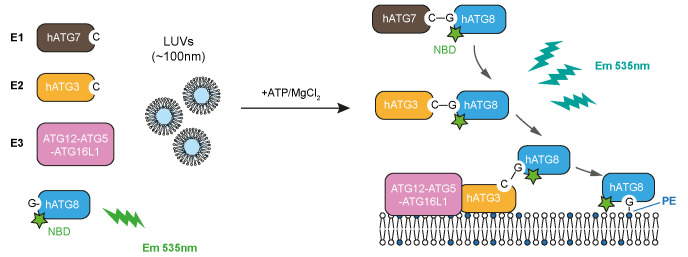


**Experimental design to track the full reaction of ATG8 lipidation, described in this protocol**

## Background

Autophagy is a well-conserved bulk degradation pathway from yeast to mammals. It occurs ubiquitously in response to metabolic demands and plays an important role in maintaining cellular homeostasis and cell survival (Mizushima and Komatsu, 2011). Upon autophagy induction, cytosolic materials are sequestered and enclosed by a double-membrane structure, called an autophagosome, leading to the degradation of the content after fusion with lysosomes. One of the key discoveries in autophagy field is ATG8 lipidation via the ubiquitin-like conjugation systems (Mizushima, 2020; Nishimura and Tooze, 2020). The ubiquitin-like ATG8 is first primed by a cysteine protease ATG4 to expose its C-terminal glycine. Then, the activated ATG8 is conjugated to ATG7 (E1) in an ATP-dependent manner and transferred to ATG3 (E2). It is finally covalently conjugated to the headgroup of phosphatidylethanolamine (PE), catalysed by ATG12–ATG5-ATG16L1 complex (hereafter, the E3 complex). Lipidated ATG8 is commonly used as autophagosomal membrane marker, and ATG8 lipidation is monitored to assess the autophagy activity in cells (Mizushima et al., 2010).

In vitro reconstitution of ATG8 lipidation with synthetic membrane models, such as large unilamellar vesicles (LUVs), is also well established (reviewed in Huang et al., 2022). The end-point level of lipidated ATG8, which is usually examined by SDS-PAGE, has been used to assess the function of ATG7 (Taherbhoy et al., 2011; Noda et al., 2011), ATG3 (Nath et al., 2014), and the E3 complex (Lystad et al., 2019). However, this end-point readout cannot track ATG8 conjugation reactions in a real-time manner or provide more information on the reaction rate.

Recently, we designed a real-time lipidation assay using human ATG8 proteins (LC3B/GABARAP) N-terminally labelled with 7-nitrobenz-2-oxa-1,3-diazol-4-yl (NBD) (Zhang et al., 2023). As NBD fluoresces brightly around 535 nm when it is located in a hydrophobic environment, we use it to dynamically track the hydrophobic environment that ATG8 N-terminus encounters during the lipidation reaction (provided by the ATG7 or ATG3 interfaces and/or the membrane). Interestingly, the increased NBD signals in the real-time assay, in addition to responding to membrane environments, also reflect the enzymatic activity of ATG7, ATG3, and the E3 complex, which can be readily adapted to evaluate the function of ATG7, ATG3, the E3 complex, or other upstream regulators, and provide a kinetic measurement in future studies. This protocol provides the setup for the real-time lipidation assay using NBD-labelled LC3B, as an example.

## Materials and reagents

1. Sf9 cells (ATCC, catalog number: CRL-1711)
2. BL21 (DE3) *E. coli* (New England BioLabs, catalog number: C2527H)
3. Sf-900^TM^ II SFM medium (Thermo Fisher Scientific, catalog number: 10902104)
4. Fugene HD transfection reagent (Promega, catalog number: E2311)
5. flashBAC GOLD (Oxford Expression Technologies, catalog number: 100202)
6. Gentamicin (10 mg/mL) (Thermo Fisher Scientific, catalog number: 15710049)
7. Amphotericin B (Thermo Fisher Scientific, catalog number: 15290018)
8. Centrifugal filters (0.2 μm) (VWR, catalog number: 516-0233)
9. Ampicillin (Sigma-Aldrich, catalog number: A9518-25G)
10. Kanamycin (Sigma-Aldrich, catalog number: K4000-25G)
11. Isopropyl-β-D-thio-galactopyranoside (IPTG) (Melford, catalog number: I56000-25.0)
12. Tris Base (Fisher Scientific, catalog number: BP1521)
13. Hydrochloric acid (HCl), 37% (Fisher Scientific, catalog number: 10053023)
14. Sodium chloride (NaCl) (Fisher Scientific, catalog number: 15526005)
15. Tris(2-carboxyethyl)phosphine hydrochloride solution (TCEP) (Sigma-Aldrich, catalog number: 646547-10X1ML)
16. AEBSF hydrochloride (AppliChem, catalog number: A1421)
17. Benzamidine (Sigma-Aldrich, catalog number: B6506-25G)
18. cOmplete^TM^, EDTA-free protease inhibitor cocktail (Sigma-Aldrich, catalog number: 11873580001)
19. Glutathione Sepharose^®^ 4B beads (Cytiva, catalog number: 17075605)
20. IANBD (Invitrogen, catalog number: D2004)
21. Dimethyl sulfoxide (DMSO) (Sigma-Aldrich, catalog number: D2650)
22. L-cysteine (Sigma-Aldrich, catalog number: 168149)
23. Glycerol (Sigma-Aldrich, catalog number: 2025)
24. 100 mM MgATP (R&D Systems, Inc, catalogue number B-20)

**Lipids**

1. 16:0-18:1 PC (POPC) (Avanti Polar Lipids, catalog number: 850457C)
2. 18:1 (Δ9-Cis) PE (DOPE) (Avanti Polar Lipids, catalog number: 850725C)

**Solutions**

1. Lysis buffer 1 (see Recipes)
2. Lysis buffer 2 (see Recipes)
3. Wash buffer (see Recipes)
4. Equilibration buffer (see Recipes)
5. Labelling buffer (see Recipes)
6. Assay buffer (see Recipes)

**Recipes**

1. **Lysis buffer 1**
  50 mM Tris-HCl, pH 8.0
  500 mM NaCl
  0.5 mM TCEP
  0.4 mM AEBSF
  15 μg/mL benzamidine
  Store at 4 °C, use within three days.
2. **Lysis buffer 2**
  50 mM Tris-HCl, pH 8.0
  500 mM NaCl
  0.5 mM TCEP
  1× cOmplete^TM^, EDTA-free protease inhibitor
  Store at 4 °C, use within three days.
3. **Wash buffer**
  50 mM Tris-HCl, pH 8.0
  500 mM NaCl
  0.5 mM TCEP
  Store at 4 °C, use within two weeks.
4. **Equilibration buffer**
  25 mM Tris-HCl, pH 8.0
  150 mM NaCl
  0.5 mM TCEP
  Store at 4 °C, use within two weeks.
5. **Labelling buffer**
  25 mM Tris-HCl, pH 7.5
  150 mM NaCl
  Store at 4 °C, use within two weeks.
6. **Assay buffer (the same as equilibration buffer)**
  25 mM Tris-HCl, pH 8.0
  150 mM NaCl
  0.5 mM TCEP
  Store at 4 °C, use within two weeks.

## Equipment

1. Sonicator (MSE, model: Soniprep 150)
2. Vivaspin 20 concentrator column (MWCO 10K) (Cytiva, catalog number: 28932360)
3. Vivaspin 6 concentrator column (MWCO 30K) (Cytiva, catalog number: 28932317)
4. ӒKTA pure^TM^ equipped with Superdex 200 16/60 GL column (Cytiva, catalog number: 28-9893-35) and Superdex 200 10/300 GL column (Cytiva, catalog number: 17-5175-01)
5. PD Minitrap^TM^ G-25 (Sigma-Aldrich, catalog number: GE28-9180-07)
6. Extruder set with holder/heating block including mini-extruder, two syringes, polycarbonate membranes 0.1 µm 19 mm, 100 filter supports, and one holder/heating block (Avanti Polar Lipids, catalog number: 610000-1EA)
7. Polycarbonate membranes 0.2 μm 19 mm (Avanti Polar Lipids, catalog number: 610006-1EA)
8. Eppendorf^TM^ Concentrator Plus (Eppendorf, catalog number: 5305000568)
9. Zetasizer Nano ZS (Malvern Instruments)
10. Ultra-micro cuvette 10 mm pathlength, 100 μL (Hellma Analytics, catalog number: 105.201-QS)
11. Spectrofluorometer (JASCO, model: FP-8300)
12. Ti45 rotor (Beckman, 339160)

## Software and datasets

1. Excel (Microsoft Office, https://www.office.com/)
2. GraphPad Prism 9 (GraphPad Software, https://www.graphpad.com/)

## Procedure

- **ATG3 expression and purification from BL21 (DE3) *E. coli***
  1. Transform pGEX6P1-GST-3C-ATG3 into BL21 (DE3) *E. coli* cells, spread the bacteria on an LB agar plate containing 100 μg/mL of ampicillin, and incubate the plate at 37 °C overnight.
  2. Inoculate a single colony into 120 mL of LB medium containing 100 μg/mL ampicillin in a 250 mL glass flask and incubate at 37 °C shaking at 180 rpm overnight in a shaking incubator.
  3. Dilute 25–30 mL of the culture to 1 L of the LB medium containing 100 μg/mL ampicillin in a 2 L glass flask (OD_600_ around 0.1). In total, prepare 4 L of culture. Grow the culture for 3–5 h at 37 °C shaking at 200 rpm in a shaking incubator, until OD_600_ reaches 0.8.
  4. Induce protein expression by adding 500 μL of 1 M IPTG (final concentration 0.5 mM) and incubate at 18 °C shaking at 200 rpm in a shaking incubator overnight.
  5. Pellet the bacteria at 4,000 rpm for 15 min and discard the medium.
  6. Resuspend the bacteria pellet in 50 mL of lysis buffer 1 and directly place in a -20 °C freezer for at least one day to break the cells. This is an optional step; the aim is to break the bacteria through gradual ice crystal formation.
  7. Thaw the bacteria pellets in ice-cold water and sonicate the pellet on ice at maximum amplitude of 20 μm for 40 s, interval 60 s. Repeat 5–10 times until the cell lysate becomes less viscous.
  8. Pellet cell debris using the Ti 45 rotor at 25,000× *g* at 4 °C for 30 min.
  9. Collect supernatant and add 4 mL of Glutathione Sepharose^®^ 4B slurry (i.e., 2 mL of Glutathione Sepharose^®^ 4B beads). Incubate rolling at 4 °C for 90 min.
  10. Pellet the beads at 500× *g* for 3 min at 4 °C. Wash the beads with wash buffer five times.
  11. Resuspend the beads in 10 mL of wash buffer, add 200 μL of GST-3C (5 mg/mL, obtained from Crick Structural Biology Platform), and incubate rolling at 4 °C overnight. GST-3C protease is also commercially available, and can be purchased from Merck (PreScission Protease, catalog number: GE27-0843-01).
  12. Collect eluate and concentrate the protein with a Vivaspin 20 concentrator column (MWCO 10K) until the volume reaches 1–2 mL.
  13. Further purify the protein by size exclusion chromatography (SEC) using the Superdex 200 16/60 GL column equilibrated in equilibration buffer.
  14. Check the protein purity by SDS-PAGE, collect the peak fraction, and concentrate the protein using a Vivaspin 20 concentrator column (MWCO 10K). Final stock protein concentration is usually ~6 mg/mL.
  15. Flash freeze in liquid N_2_ and store at -80 °C until use.
- **LC3B S3C expression and purification from BL21 (DE3) *E. coli***
  1. Transform pAL-GST-3C-LC3B_(1-120)_ S3C construct into BL21 (DE3) *E. coli* cells, spread the bacteria on an LB agar plate containing 50 μg/mL of kanamycin, and incubate the plate at 37 °C overnight.
  2. Inoculate a single colony into 120 mL of LB medium containing 50 μg/mL kanamycin in a 250 mL glass flask and incubate at 37 °C shaking at 180 rpm overnight in a shaking incubator.
  3. Dilute 25–30 mL of the culture to 1 L of the LB medium containing 50 μg/mL kanamycin in a 2 L glass flask (OD_600_ around 0.1). In total, prepare 4 L of culture. Grow the culture for 3–5 h at 37 °C shaking at 200 rpm in a shaking incubator, until OD_600_ reaches 0.8.
  4. Induce protein expression by adding 500 μL of 1 M IPTG (final concentration 0.5 mM) and incubate at 18 °C shaking at 200 rpm in a shaking incubator overnight.
  5. Pellet the bacteria at 4,000 rpm for 15 min and discard the medium.
  6. Resuspend the bacteria pellet in 50 mL of lysis buffer 1 and directly place in a -20 °C freezer for at least one day to break the cells. This is an optional step; the aim is to break the bacteria through gradual ice crystal formation.
  7. Purification steps are performed as described in section A (steps 7–14). Final stock concentration is usually ~7 mg/mL.
  8. Flash freeze in liquid N_2_ and store at -80 °C until use.
*Note: This purification protocol was also used to purify other ATG8 proteins.*
- **ATG7 expression and purification from insect cells Sf9**
  1. Filter the transfer plasmid pBacPAK-His_3_-GST-ATG7 with centrifugal filters (0.2 μm) to sterilise before use.
  2. Seed 0.5 × 10^6^ Sf9 cells per well in a 6-well plate and incubate in a static incubator at 27 °C for at least 1 h.
  3. Prepare the transfection mix: add 0.5 μg of the transfer plasmid and 50 ng of flashBAC GOLD DNA into 200 μL of Sf-900^TM^ II SFM medium. Then, add 2 μL of Fugene HD transfection reagent. Incubate at room temperature for 20 min.
  4. Add 800 μL of Sf-900^TM^ II SFM medium to the transfection mix.
  5. Aspirate the medium from the cells in the 6-well plate and add ~1 mL of the mixture on the top of cells. Incubate in a static incubator at 27 °C overnight.
  6. Add another 1 mL of Sf-900^TM^ II SFM medium containing gentamicin (10 μg/mL) and amphotericin B (0.25 μg/mL) on the top of the cells in the 6-well plate.
  7. Incubate for another four days in a static incubator at 27 °C. Harvest and pool the cells and supernatant and add 1.5 mL of the cell mixture into 50 mL of S9 cells at a density of 1 × 10^6^ cells/mL. Incubate at a shaking incubator at 27 °C and 140 rpm for three days.
  8. Collect the supernatant, which contains baculovirus harbouring His_3_-GST-ATG7 construct.
*Note: The successful production of baculovirus harbouring His_3_-GST-ATG7 construct was checked by immunoblotting ATG7 or the GST-tag in the cell pellet.*
  9. Culture 400 mL of Sf9 cells in Sf-900^TM^ II SFM medium to a density of 1.5 × 10^6^ cells/mL and add 1 mL of baculovirus harbouring His_3_-GST-3C-ATG7 construct. Incubate in a shaking incubator at 27 °C and 140 rpm.
  10. After 60 h of infection, harvest the cells by centrifugation at 600× *g* for 10 min at 4 °C.
  11. Freeze at -80 °C until ready for purification.
  12. Thaw the cell pellet on ice, resuspend it with 30 mL of lysis buffer 2, and sonicate the mixture on ice at 50% amplitude for 10 s, interval 60 s. Repeat 3–5 times.
  13. Pellet cell debris using the Ti 45 rotor at 30,000× *g* for 30 min at 4 °C.
  14. Collect supernatant and add 2 mL of Glutathione Sepharose^®^ 4B slurry (i.e., 1 mL of Glutathione Sepharose^®^ 4B beads). Incubate rolling at 4 °C for 90 min.
  15. Pellet the beads at 500× *g* for 3 min at 4 °C. Wash the beads with wash buffer five times.
  16. Resuspend the beads in 5 mL of wash buffer, add 100 μL of GST-3C (5 mg/mL, obtained from Crick Structural Biology Platform), and incubate rolling at 4 °C overnight.
  17. Collect eluate and concentrate the protein with a Vivaspin 6 concentrator column (MWCO 30K) until the volume reaches 300–500 μL.
  18. Further purify the protein by SEC using the Superdex 200 10/300 GL column equilibrated in equilibration buffer.
  19. Check the protein purity by SDS-PAGE, collect the peak fraction, and concentrate the protein using a Vivaspin 6 concentrator column (MWCO 30K). Final stock concentration is ~2 mg/mL.
  20. Flash freeze in liquid N_2_ and store at -80 °C until use.
- **NBD-labelling of ATG8 proteins with single cysteine mutation (LC3B S3C)**
  1. Prepare 10 mM IANBD-amide stock dissolved in DMSO. For longer storage, keep the stock at -20 °C.
  2. Equilibrate a PD Minitrap G-25 column with 8 mL of labelling buffer. Apply 250 μL of 5 mg/mL LC3B S3C to the column and collect the eluate.
*Note: Alternative sources of desalting columns or other buffer exchange methods such as dialysis can be used as well.*
  3. After buffer exchange, re-measure the protein concentration, prepare 400 μL of ~100 μM LC3B S3C, and mix with 100 μL of 10 mM IANBD-amide. Incubate the reaction mix at room temperature for 1 h in the dark.
  4. Add 20 μL of 100 mM L-cysteine to quench the reaction.
  5. Equilibrate another PD Minitrap G-25 column with 8 mL of assay buffer. Apply ~500 μL of the reaction mix on the column and collect the eluate. This step removes the excess IANBD-amide.
  6. Measure protein concentration by Bradford assay and run SDS-PAGE to check the NBD labelling and if there is no excess dye (unreacted dye migrates in dye front).
  7. Mix 125 μL of glycerol with 500 μL of NBD-labelled LC3B S3C to a final concentration of 20%. Aliquot the protein, flash freeze in liquid N_2_, and store at -80 °C.
- **Liposome preparation**
  To prepare liposome stock (1 mL, 2 mM final concentration)
  1. Mix POPC and DOPE lipids at a ratio of 50:50 (% mol).
  2. Dry the lipids under nitrogen gas for 5 min and further dry in the Eppendorf^TM^ Concentrator for 2 h.
  3. Add 1 mL of assay buffer onto the lipid film and vortex thoroughly until the lipid film is fully resuspended.
  4. Conduct five freeze-thaw cycles in liquid nitrogen and 42 °C water bath until the lipid solution is fully thawed.
  5. Prepare the unilamellar vesicles with the Mini Extruder. Extrude the liposome solution first by passing the solution 21 times through a 0.2 μm membrane. Then, extrude the solution by passing it at least 41 times through a 0.1 μm membrane.
*Note: It is recommended to extrude the liposomes through a 0.2 μm membrane before a 0.1 μm membrane in order to make homogeneous liposomes.*
  6. Check the liposome size by Zetasizer Nano ZS [for example, Figure 1: average size 105.5 nm, PDI (polydispersity index) = 0.071]. If the size of liposomes is not homogeneous (PDI > 0.2), repeat the extrusion step through the 0.1 μm membrane.
    **Figure 1.**
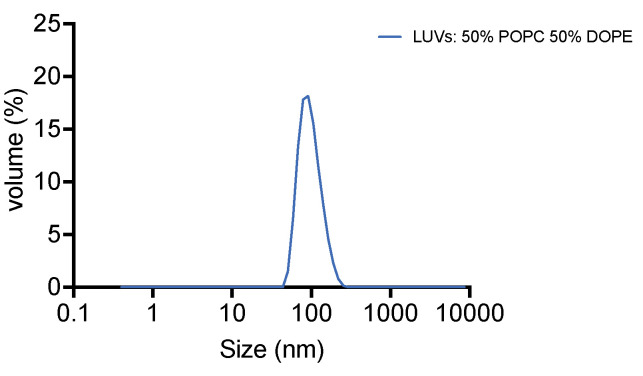
Result of liposome size distribution measured by dynamic light scattering [polydispersity index (PDI) = 0.071]
  7. Keep the liposomes at 4 °C and use within two days.
- **Real-time ATG8 lipidation assay**
  1. Purified ATG7, ATG3, LC3B S3C, and LC3B S3C^NBD^ are resolved by SDS-PAGE (Figure 2).
    **Figure 2.**
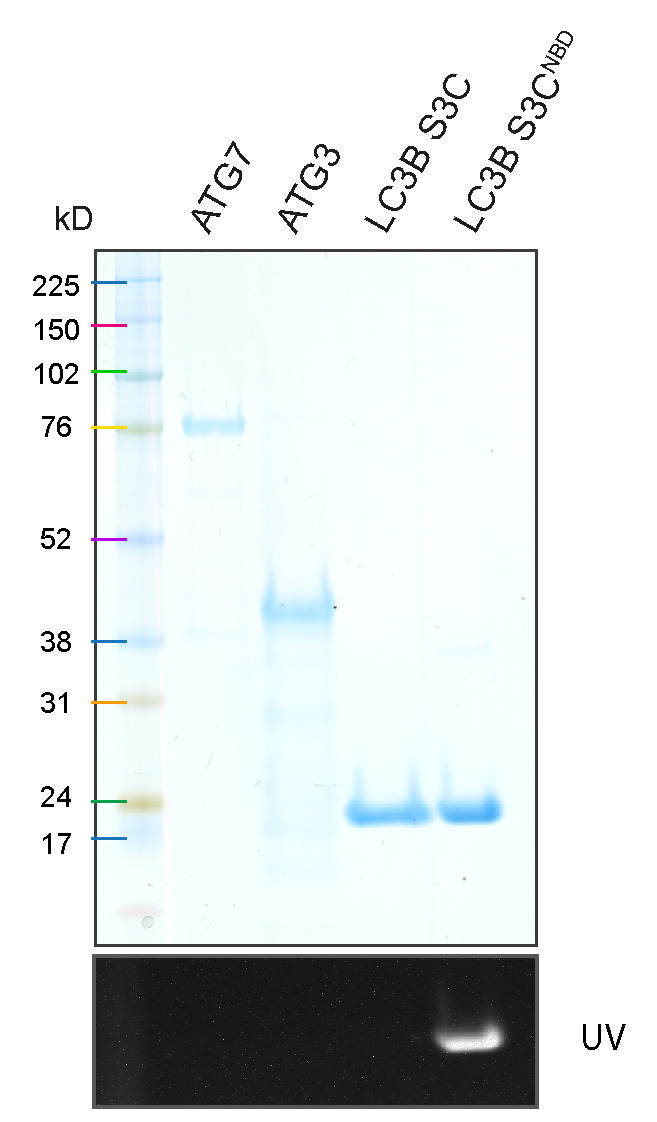
SDS-PAGE result of purified ATG7, ATG3, LC3B S3C, and NBD-labelled LC3B S3C
  2. Table 1 indicates the concentration and volume for each component in the real-time lipidation ATG8 assay.
    **Table 1.** Components for the real-time ATG8 lipidation assay (total volume: 78.4 μL)

Component	Final concentration	Volume to add (stock concentration)
ATG7	0.2 μM	0.8 μL (20 μM)
ATG3	0.2 μM	0.8 μL (20 μM)
E3 complex Liposomes LC3B S3C^NBD^	0.05 μM 1 mM 1 μM	0.2 μL (20 μM) 40 μL (2 mM) 1.6 μL (50 μM)
Assay buffer	n/a	35 μL

*Note: To characterise the effect of each component on the real-time assay, add the amount of buffer instead of the component of interest. For example, for “no ATG7” condition, prepare the reaction mix without ATG7 and add buffer instead. The protein stock can be diluted to a lower concentration so that it will be easier to pipette a larger volume of protein.*
  3. Transfer the reaction mix into a 10 mm path length quartz cuvette (100 μL).
  4. Record NBD fluorescence (ex/em 468 nm/535 nm) over time using a FP-8300 spectrofluorometer.
  5. Set cuvette holder temperature to 37 °C.
  6. Set excitation at 468 nm (bandwidth 5 nm) and emission at 535 nm (bandwidth 10 nm).
  7. Set the total measurement time to 20 min with two measurement ranges:
    Time interval 1: 0–80 s; record NBD fluorescence every 20 s (equilibration step before the reaction).
    Time interval 2: 80–1,200 s; record NBD fluorescence every 10 s (measurement step after adding ATP).
  8. Start the measurement. By the end of time interval 1 (between 60 and 80 s), add 1.6 μL of MgATP (50 mM) to the reaction (final concentration of MgATP: 1 mM) and mix properly.
  9. Repeat these steps for each condition: *no ATG7, no ATG3, no E3, no liposomes, no ATP*, and *All*.
*Note: With “no ATP” condition, add 1.6 μL of assay buffer instead between 60 and 80 s (the end of time interval 1).*

## Data analysis

1. The NBD fluorescence increase *ΔEm535 nm* at each time point in the reaction groups was calculated by subtracting the NBD fluorescence recorded from control group (i.e., *no ATP* condition), as shown in Figure 3A.
$$∆Em535\;nm\;(t)=F_{Reaction}\left(t\right)-F_{"no\;ATP"}\left(t\right)$$
2. The relative fluorescence *ΔF* at each time point was normalised to the time point at 80, as shown in Figure 3B.
$$∆Em535\;nm\;(t=80s)=F_{Reaction}\left(t=80s\right)-F_{"no\;ATP"}\left(t=80s\right)$$
$$∆F\left(t\right)=∆Em535\;nm\;\left(t\right)-∆Em535\;nm\;(t=80s)$$
3. Analyse the data in Excel and plot data in Prism 9.0.
  **Figure 3.**
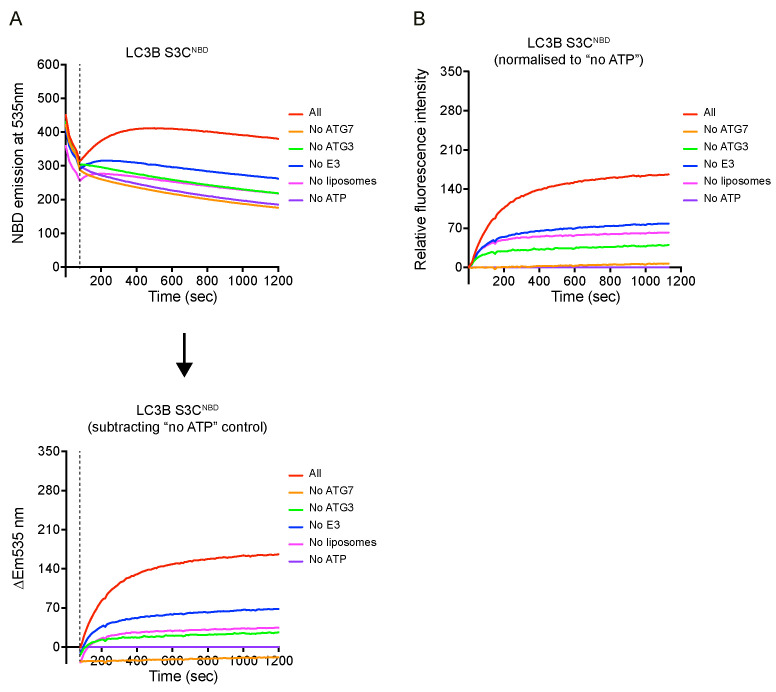
Example of the real-time lipidation assay. (A) Raw data of NBD spectra over time. After adding ATP between 60 and 80 s, signals were increased except for “no ATG7” and “no ATP” conditions. The NBD signal increase (*ΔEm535 nm*) was calculated by subtracting the NBD signal from the control group *no ATP* condition from the time point 80 s. (B) Relative fluorescent increase normalised to *no ATP* condition at time point 80 s. One repeat of three independent experiments is shown here.

## Validation of protocol

This protocol was used in Zhang et al. (2023), DOI: 10.7554/eLife.89185

## General notes and troubleshooting

1. The E3 complex used in this protocol was purified by Anne Schreiber (The Francis Crick Institute), as previously described in Zhang and Nishimura et al. (2023). The protein purification protocol for the in vitro ATG8 lipidation reaction has been described previously, for example Landajuela et al. (2016), Zheng et al. (2017), Lystad et al. (2019), and Fracchiolla et al. (2020).
2. This protocol employed a simple lipid model, which is ~100 nm liposomes containing 50% PC and 50% PE. To further assess the effects of specific lipids or membrane curvature on ATG8 lipidation reaction, the lipid composition and liposome size can be modified, respectively, according to the experimental purpose.
